# Brain stereotactic radiosurgery using MR‐guided online adaptive planning for daily setup variation: An end‐to‐end test

**DOI:** 10.1002/acm2.13518

**Published:** 2022-01-07

**Authors:** Eun Young Han, He Wang, Tina Marie Briere, Debra Nana Yeboa, Themistoklis Boursianis, Georgios Kalaitzakis, Evangelos Pappas, Pamela Castillo, Jinzhong Yang

**Affiliations:** ^1^ Department of Radiation Physics The University of Texas MD Anderson Cancer Center Houston Texas USA; ^2^ Department of Radiation Oncology The University of Texas MD Anderson Cancer Center Houston Texas USA; ^3^ Department of Medical Physics University of Crete Heraklion Greece; ^4^ Department of Biomedical Sciences, Radiology and Radiotherapy Sector University of West Attica Athens Greece

**Keywords:** adaptive planning, brain metastases, end‐to‐end test, MR Linac

## Abstract

Online magnetic resonance (MR)‐guided radiotherapy is expected to benefit brain stereotactic radiosurgery (SRS) due to superior soft tissue contrast and capability of daily adaptive planning. The purpose of this study was to investigate daily adaptive plan quality with setup variations and to perform an end‐to‐end test for brain SRS with multiple metastases treated with a 1.5‐Tesla MR‐Linac (MRL). The RTsafe PseudoPatient Prime brain phantom was used with a delineation insert that includes two predefined structures mimicking gadolinium contrast‐enhanced brain lesions. Daily adaptive plans were generated using six preset and six random setup variations. Two adaptive plans per daily MR image were generated using the adapt‐to‐position (ATP) and adapt‐to‐shape (ATS) workflows. An adaptive patient plan was generated on a diagnostic MR image with simulated translational and rotational daily setup variation and was compared with the reference plan. All adaptive plans were compared with the reference plan using the target coverage, Paddick conformity index, gradient index (GI), Brain V12 or V20, optimization time and total monitor units. Target doses were measured as an end‐to‐end test with two ionization chambers inserted into the phantom. With preset translational variations, V12 from the ATS plan was 17% lower than that of the ATP plan. With a larger daily setup variation, GI and V12 of the ATS plan were 10% and 16% lower than those of the ATP plan, respectively. Compared to the ATP plans, the plan quality index of the ATS plans was more consistent with the reference plan, and within 5% in both phantom and patient plans. The differences between the measured and planned target doses were within 1% for both treatment workflows. Treating brain SRS using an MRL is feasible and could achieve satisfactory dosimetric goals. Setup uncertainties could be accounted for using online plan adaptation. The ATS workflow achieved better dosimetric results than the ATP workflow at the cost of longer optimization time.

## 1 INTRODUCTION

Brain metastases are the most common intracranial tumors, and 30%–40% of cancer patients could develop brain metastases during the course of their disease.^1^ Stereotactic radiosurgery (SRS) and fractionated stereotactic radiotherapy (SRT) using precisely focused high‐dose radiation are increasingly used to treat single and multiple brain metastases due to concerns about the neurocognitive effects from whole‐brain radiation treatment.^2^, ^3^, ^4^ Pretreatment daily cone beam CT (CBCT) scans are typically used to align the patient's skull as a surrogate to verify target positioning.^5^, ^6^ Unfortunately, metastatic brain lesions are normally undetectable on routine CBCT scans. The preferred imaging for brain metastases is T1 contrast‐enhanced magnetic resonance imaging (MRI).**^7^**

The recent innovation of MR‐guided radiotherapy (MRgRT) may provide a promise for the treatment of brain metastases. With MRgRT, an MRI unit is integrated with a linear accelerator to provide real‐time imaging of the target and organ‐at‐risk (OAR) volumes before and during treatment delivery. Online plan adaptation is available with an MRgRT system with an integrated treatment planning system. Cao et al. reported that the superior soft tissue contrast with MRgRT enables better identification of targets and OARs and better motion management of selected tumor sites. In addition, the functional MR imaging will also extend the ability to develop individualized and real‐time adaptation strategies.^8^ Recently, the Elekta Unity MR‐guided Linac (MRL, Elekta, Stockholm,Sweden), integrating a 1.5‐Tesla Philips MRI system (Amsterdam, Netherlands), an Elekta 7‐MV Linac, and Monaco treatment planning system (Elekta, Stockholm, Sweden), was approved for clinical use in our institution. The characteristics of the Unity MRL include 7.2‐mm leaf width multi‐leaf collimator (MLC), 143.5 cm SAD, and coplanar sagittal MLC beams for step‐and‐shoot intensity‐modulated radiation therapy (IMRT) planning.

The feasibility of stereotactic body radiotherapy (SBRT) using the MRL system has been evaluated in various treatment sites.^9^, ^10^, ^11^, ^12^, ^13^, ^14^ The MRL is also expected to benefit brain SRS due to on‐board soft tissue contrast and the capability of daily adaptive planning. Tseng et al. reported that it is feasible to generate stereotactic radiation plans that satisfy clinical requirements using MRL in the setting of a single brain metastasis.^10^ The authors reported that the dosimetric impact of the magnetic field including the electron return effect at the tissue–air boundaries is minor and does not negatively impact the target conformity or dose gradient.^10^ A study by Ruschin et al. showed for translational setup variation in brain SRS on the MRL, the virtual couch shift (VCS) method^15^, ^16^ is an acceptable correction strategy. The authors demonstrated that translational variation can be corrected with the VCS method by simulating known translational variation of varying magnitudes and orientations.^15^ However, the feasibility of treating brain SRS patients with multiple brain metastases has not been investigated with regard to daily adaptive plan quality using the adapt‐to‐position (ATP) and adapt‐to‐shape (ATS) planning workflows used in Unity MRL.

The purpose of the present study was to evaluate the adaptive treatments for brain SRS on the MRL impacted by daily setup variation. Daily adaptive plans were generated for the MR images of a head phantom and a patient using the ATP and ATS workflows with preset or random daily setup variations and compared with the reference plan. Target doses were measured with two ionization chambers for both workflows in an end‐to‐end test.

## 2 MATERIALS AND METHODS

### 2.1 Head phantom with delineation insert

**FIGURE 1:**
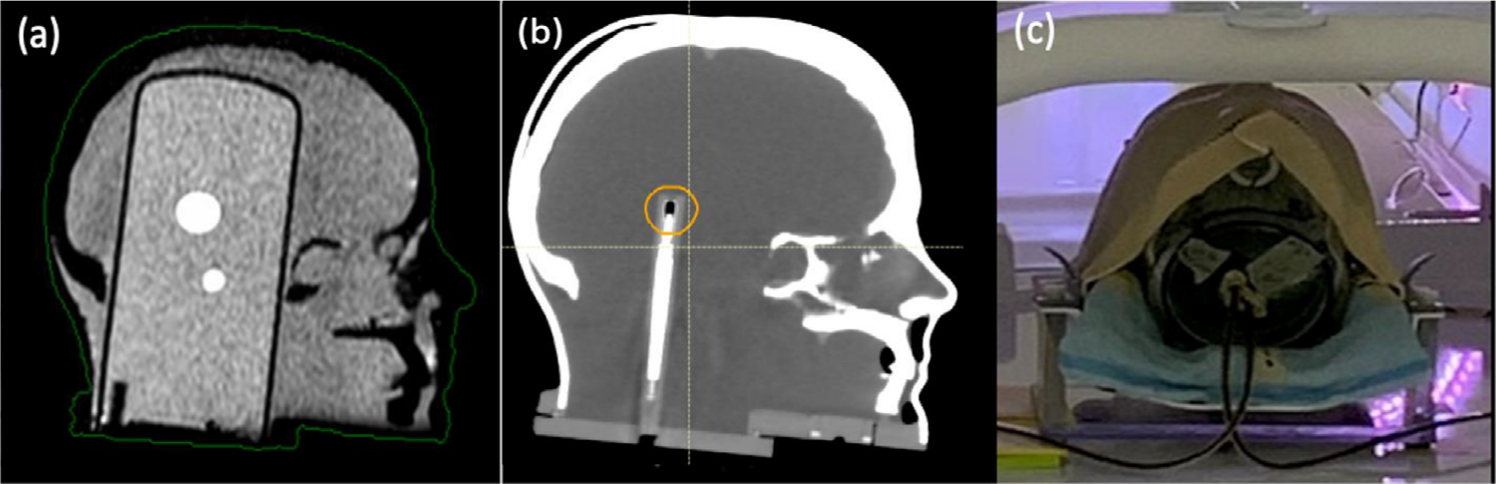
(a) Sagittal plane of T1w magnetic resonance (MR) images of the Prime head phantom with a delineation insert containing two brain lesions. (b) Sagittal plan of CT images of the same phantom with the chamber insert (one chamber shown at this cross‐section with chamber head contoured). (c) Measurement setup with two chambers

A PseudoPatient Prime head phantom (RTsafe, Athens, Greece) was used in this study (Figure 1). The phantom is a 3D‐printed anatomical replica created from a CT image of a human head. The hollow phantom with internal anatomical bony structures can be filled with water and has an insert to hold an ionization chamber for point dose measurement.^17^ Recently, RTsafe developed a new delineation insert that fits into their Prime phantom with two predefined brain metastatic lesions near brainstem that are visible on MRI (Figure 1a). The superior target has a volume of 4.8 cm^3^, the inferior target has a volume of 1.4 cm^3^, and the density of both targets is 1.03 g/cm^3^. The superior target is located 3.0 cm above the center of inferior target. The cylinder insert is made of a tissue equivalent material. In order to measure target doses, a customized ionization chamber insert consisting of two polymethyl methacrylate (PMMA) plugs with 2.8‐mm wall thickness was manufactured to fit two Exradin A1SLMR ionization chambers (Figure 1b).

### 2.2 Reference plan creation

The head phantom was immobilized using a Moldcare head cushion (Alcare, Tokyo, Japan) and a thermoplastic mask (Orfit Industries, Norfolk, VA), as shown in Figure 1c. Reference CT images were acquired with the delineation insert using a Phillips Brilliance BigBore CT scanner (Phillips, Amsterdam, Netherlands) with a slice thickness of 1 mm. Reference MR images were acquired using the MRL with 1‐mm slice thickness. The superior gross tumor volume (Sup GTV) and inferior gross tumor volume (Inf GTV) were defined by delineating the enhanced lesions on T1‐weighted MR images. A planning target volume (PTV) margin of 1 mm was applied to each GTV. The two sensitive volumes from the chamber insert were outlined inside the target volumes on the CT image. All regions of interest (ROIs) were contoured on CT images with the reference MR image fused.

The reference plan was created following clinical goals for SRS to achieve 100% coverage of the GTVs (*D*_min_ ≥20 Gy, single fraction) and at least 95% coverage of the PTVs with 95% of the prescription dose (V95% ≥95%). A dose maximum (*D*_max_) of less than 115% of the prescription was allowed within the PTVs. A step‐and‐shoot IMRT plan was generated with the Monaco treatment planning system (v5.4, Elekta AB, Stockholm, Sweden). Thirteen co‐planar beam angles (196°, 224°, 265°, 278°, 308°, 336°, 0°, 28°, 56°, 84°, 100°, 140°, and 168°) were used. Dose calculations were performed with the GPU‐oriented Monte Carlo dose calculation algorithm (GPUMCD),^18^ with a statistical uncertainty of <1% per total plan, 2 mm of dose calculation grid size, a minimum of 5 monitor units (MU) per segment, and a minimal segment width of 0.5 cm.

### 2.3 Adaptive plan creation

All MRL treatments use a plan adaptation process to account for daily setup variability. The Monaco treatment planning software provides two different plan adaptation workflows: “adapt‐to‐position” (ATP) and “adapt‐to‐shape” (ATS). In the ATP process, no daily structure delineation is needed. The daily MRI scan is used to identify the translational isocenter shift from the reference plan position using translation‐only registration. The shape and weight of the beam segments in the reference plan are optimized on the reference image to match the daily position of targets and OARs. The same dose constraints and optimization parameters are used as in the reference plan. In the ATS process, deformable image registration is performed to propagate all contours from the reference image to the daily MR image. The deformed target and critical OARs are then reviewed and/or manually adjusted by a radiation oncologist before adaptive planning. The daily adaptive plan can be optimized from fluence map optimization based on the anatomy on the daily MRI images, and the concluding segments may be completely different from the those in reference plan. In addition, IMRT objectives can be adjusted in ATS workflow when needed. Dose calculations are performed on a corresponding synthetic CT that matches the anatomy on the daily MR image.

#### 2.3.1 Adaptive plans created from preset daily setup variations

According to previous studies^6^, ^19^, ^20^ and our unpublished clinical data, the maximum translational and rotational pretreatment setup variation most likely lies within 3 mm and 3° for brain or head‐and‐neck radiosurgery treatments. Therefore, in order to evaluate daily adaptive treatment workflow, we mathematically transformed the reference MRI with +3 mm translations in the left–right (plan name X_tra), superior–inferior (Y_tra), anterior–posterior (Z_tra) directions, separately, and with +3° rotations in the pitch (X_rot), roll (Y_rot), and yaw (Z_rot) directions, separately, using MATLAB (R2014b, Mathworks, Natick, MA) to simulate daily setup variations. These six transformed MR image sets were imported into Monaco for adaptive planning, and adaptive plans were created for each of the transformed MR images using both the ATP and ATS workflows.

The ATP workflow does not correct the rotational setup variations. Thus, an nominal adaptive plan including a 3° rotation would be technically the same as the reference plan. To evaluate the difference in the actual delivered adaptive plan due to the 3^o^ rotation, the contours on the reference image were first mapped onto the rotated MR image by deformable images registration. The beams were then copied to the rotated MR image with original segments and the dose was recalculated and compared to nominal adaptive plan by the ATP workflow.

In the ATS workflow, the volumes of the daily GTVs generated by deformable image registration were recorded to track the target variations. Adaptive plans were created after all PTVs and critical structures were verified on daily MR images.

All adaptive plans were normalized so that the minimum dose criteria of both GTVs were met and were compared with the reference plan using the plan quality index, which included target coverage (PTV V95%), GTV minimum dose (*D*_min_), Paddick conformity index (PCI), gradient index (GI), brain V12 (brain volume receiving ≥12 Gy), optimization time, and total number of MU. The PCI was calculated using $\frac{{\mathrm{TV}}_{100\%}^{2}}{\mathrm{TV}\;\times PV_{100\%}}$, where TV_100%_ represents the target volume covered by prescribed dose, PV_100%_ represents the patient volume covered by the prescribed dose, and TV is the total target volume. The GI was calculated using $\frac{PV_{50\%}}{PV_{100\%}}$, where PV_50%_ represents the patient volume covered by 50% of the prescribed dose.

#### 2.3.2 Adaptive plans created from random daily setup variations

Six scans with the phantom were acquired on the MRL unit on different dates to simulate more realistic daily setup variations. The phantom was set up similarly to a real patient treatment. We varied the anterior–posterior position by removing a 9‐mm thick couch pad and the superior–inferior position by removing the index bar that usually helps with the patient setup. Two adaptive plans were generated for each scan using each of the ATP and ATS workflows, and they were compared to the reference plan using above‐mentioned plan quality index.

#### 2.3.3 Adaptive plans for simulated daily setup variations using patient images

To evaluate a realistic brain metastasis lesion with complex shape close to a brainstem, we performed a similar variation evaluation using actual patient MRI images. A patient with two right frontal brain metastasis lesions involving the basal ganglia was treated on TrueBeam sTx (Varian Medical Systems, Palo Alto, CA) using a VMAT plan with a prescription dose of 30 Gy in five fractions. A radiation oncologist contoured the GTVs and a brainstem on a planning CT fused with diagnostic MR images. PTV was generated using 2‐mm margin. These planning CT and diagnostic MR images were imported into Monaco treatment planning system to test adaptive planning workflows on patient images. All plans were created following institutional clinical goals for SRS included 100% coverage of the GTVs (*D*_min_ ≥30 Gy), at least 95% coverage of the PTVs with 95% of the prescription dose (V95% ≥95%), and brainstem V30Gy ≤0.01 cm^3^. A maximum dose (*D*_max_) of less than 115% of the prescription was allowed within the PTVs.

After a reference plan was approved by the radiation oncologist, two adaptive tests were performed to simulate the daily adaptive planning process. The planning MRI was manually transformed with (a) 3‐mm shifts in the *X*‐ and *Y*‐directions along with 3° pitch rotation (Adaptive test 1); and (b) larger shifts of 10 mm in the *X*‐ and *Y*‐directions (Adaptive test 2). Plans were generated using both ATP and ATS workflows on these two simulated MRIs, and plan quality was evaluated using the previously mentioned indices. Here we evaluated the brain V20 for this patient, instead of the brain V12 in single fraction for phantom study.

### 2.4 End‐to‐end test

For one of the random daily setups, measurements were acquired by Exradin A1SLMR (Sup GTV) and A26MR (Inf GTV) chambers (Standard Imaging, Middleton, WI). Both chambers were cross‐calibrated in a water tank with a reference chamber (PTW farmer chamber TN30013) calibrated by the ADCL (IROC, Houston, TX) in 2021. Because the chamber insert was designed for the two A1SLMR chambers, the tip of the inferior insert with the A26MR chamber was filled with water to avoid air gaps between the chamber and chamber insert and it was verified by CT imaging.

The dose to the ionization chamber was measured and compared with the mean dose to the contoured chamber‐sensitive volume in both the ATP and ATS plans. During the MR scan, the delineation insert was placed in the phantom. After adaptive plan was created and prior to beam delivery, the delineation insert was replaced carefully with the chamber insert and a verification MRI scan was performed to verify the positional consistency before and after the insert change.

## 3 RESULTS

### 3.1 Adaptive planning

**FIGURE 2:**
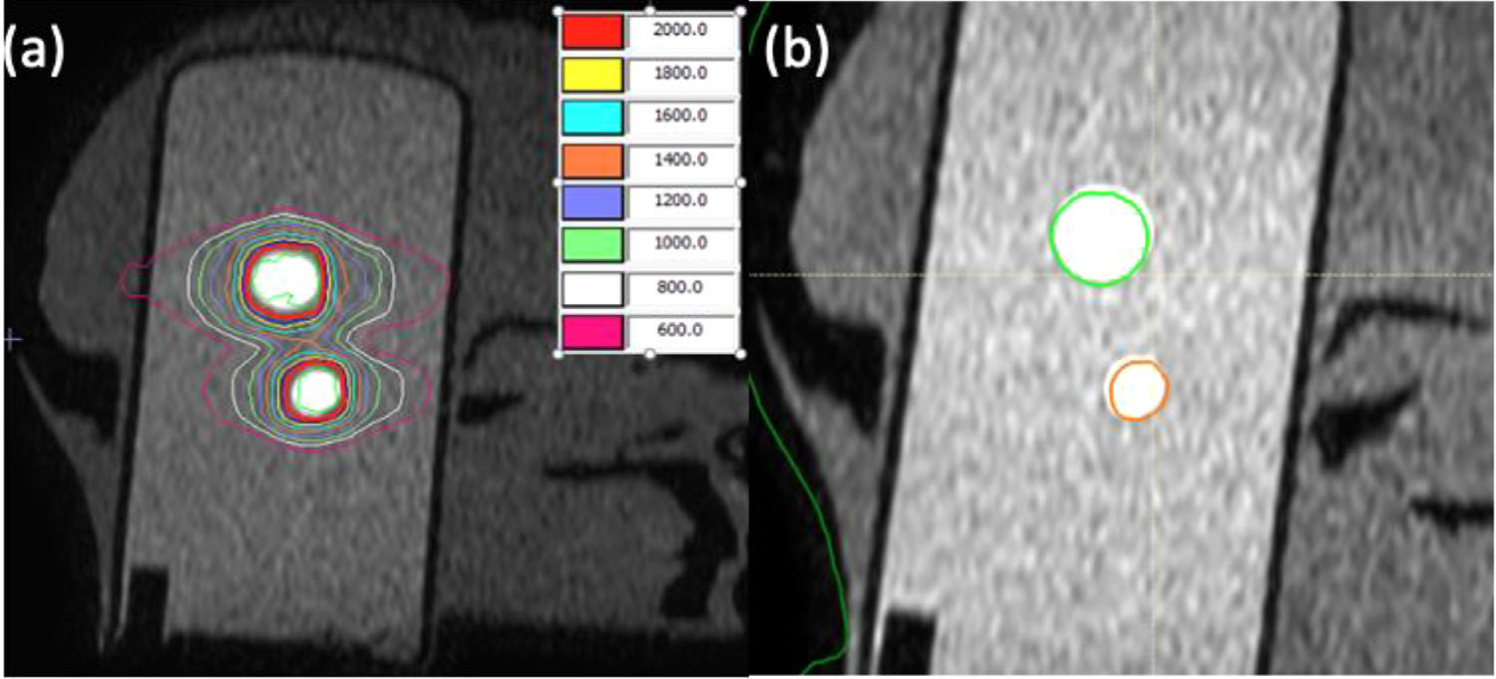
(a) Isodose lines (in cGy) in the reference plan, and (b) target volumes (green and orange contours) from the reference plan projected onto the 3° pitch‐rotated magnetic resonance imaging (MRI) by adapt‐to‐position (ATP) workflow

Figure 2a shows that the reference plan for the two targets of head phantom fulfilled the clinical goals. Image quality and the degrees of preset translation and rotation were verified with RayStation TPS (v 9A, RaySearch Laboratories, Sweden). The maximum registration errors for the preset translational and rotational shifts were 0.3 mm and 0.1^o^, respectively.

#### 3.1.1 Adaptive plans created from preset daily setup variations

**FIGURE 3:**
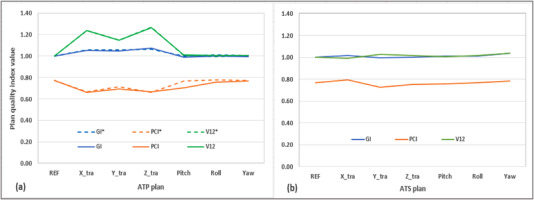
Plan quality index from (a) adapt‐to‐position (ATP) and (b) adapt‐to‐shape (ATS) workflows to daily setup variations of 3‐mm shift in lateral (X_tra), superior–inferior (Y_tra), anterior–posterior (Z_tra), and 3° rotation in pitch (X_rot), roll (Y_rot), and yaw (Z_rot), separately. In (a) the dashed lines represent the nominal ATP plans, and the solid lines represent the delivered ATP plans. GI and V12 are normalized to the values in reference plan

Figure 3 shows the plan quality index from the adaptive plans generated with preset daily setup variations, with GI and V12 normalized to the reference plan for display purpose. In ATP workflow, V12 from the nominal adaptive plans (dashed line in Figure 3a) for translational setup variations showed up to 27% increase from the reference plan, while GI showed up to 6% increase and PCI up to 14% decrease. For rotational setup variations, all indices were within ±1% of reference plan in nominal adaptive plans. In delivered adaptive plans (solid line in Figure 3a), GI and V12 remained within ±1%, while PCI decreased by 9% for 3° pitch rotation. Figure 2b shows the positional deviation between target volumes (bright area) on the 3° pitch‐rotated MRI image and the projected volume (green and orange contours) from the reference plan in the ATP workflow.

Compared to the delivered ATP plans, GI and V12 with the ATS workflow were lower by 5% and 17%, respectively, and PCI with the ATS workflow was higher by 12% for translational setup variations. The plan quality index is more consistent with the reference plan in ATS plans. Figure 3b shows that the GI, PCI, and V12 were all within ±2%–4% comparing to the reference plan. The change in GTV volume following the ATS workflow was within 0.1 cm^3^ compared to the original GTVs. Average MUs were similar regardless of workflow, but offline optimization takes more than twice as long with the ATS workflow.

Table 1 shows target coverage with preset translational and rotational setup variations following the ATP workflow. Although the target coverage met clinical goals for rotations, the delivered plans (in parenthesis) showed degradation when calculated onto the daily MR images. For daily positional deviations of 3° pitch, *D*_min_ of GTV decreased by 5% for Sup GTV, whereas for the other rotational deviations, *D*_min_ decreased up to 3% of prescription dose.

**TABLE 1:** Target coverages (V95%) and GTV mininum doses (D_min_) of ATP plans on head phantom for the setup variations of 3 mm translational shifts in lateral (X_tra), superior–inferior (Y_tra), anterior–posterior (Z_tra) directions, and 3° rotations in pitch (X_rot), roll (Y_rot), and yaw (Z_rot) directions

	**Plan name**	**REF**	**X_tra**	**Y_tra**	**Z_tra**	**X_rot**	**Y_rot**	**Z_rot**
V95% (%)	Sup PTV (delivered)	99.7	100.0	99.8	99.9	99.6	99.5	99.7
			(100.0)	(99.9)	(99.9)	(96.1)	(99.4)	(99.5)
	Inf PTV (delivered)	99.4	99.9	99.8	100.0	99.3	99.3	99.4
			(100.0)	(99.9)	(100.0)	(99.2)	(99.6)	(99.3)
*D*_min_ (cGy)	Sup GTV (delivered)	2016.6	2004	2026.4	2025.7	2032.8	2002.4	2017.9
			(2010.4)	(2003.5)	(2016.7)	**(1900.7)**	**(1947.0)**	(2010.3)
	Inf GTV (delivered)	2000.0	2000.0	2000.0	2000.0	2000.0	2000.0	2000.0
			(2033.1)	(1993.4)	(2043.7)	**(1959.6)**	**(1988.3)**	**(1982.9)**

*Note*: Numbers in the parenthesis represent the results for delivered adapt‐to‐position (ATP) plans calculated on daily image.

#### 3.1.2 Adaptive plans created from random daily setup variations

**FIGURE 4:**
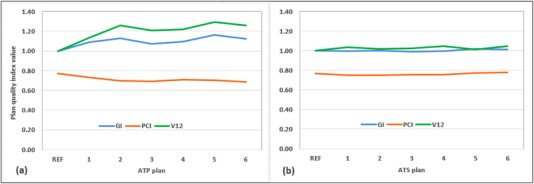
Plan quality index from (a) adapt‐to‐position (ATP) and (b) adapt‐to‐shape (ATS) workflows for six random daily setup variations

Table 2 shows the result of image registration for the six daily MRIs and reference MRI. Figure 4 shows the plan quality index from ATP and ATS workflows for random setups arranged from smaller total distance (11 mm) to larger distance (25.8 mm). As the total distance increased, the values of GI and V12 tend to increase with the ATP workflow. The average GI, V12, and PCI with the ATS workflow were 10% and 16% lower and 8% higher than those with the ATP workflow, respectively. The plan quality using the ATS workflow was again similar to the reference plan and was also more consistent among plans. The GI and V12 with the ATS workflow remained consistent within ±3% of the reference plan.

**TABLE 2:** Shifts and rotations of the head phantom relative to reference magnetic resonance imaging (MRI) obtained from image registrations of six random daily MRI setups

**Plan No**.	***X* (mm)**	***Y* (mm)**	***Z* (mm)**	**Pitch (°)**	**Roll (°)**	**Yaw (°)**	**3D (mm)**
1	1.3	10.9	0.7	2.3	0.0	−0.1	11.0
2	−3.9	11.8	0.7	1.6	0.1	3.5	12.4
3	1.9	11.7	7.1	−0.6	0.7	−0.6	13.8
4	2.0	11.9	8.9	2.1	1.3	−0.3	15.0
5	−4.4	19.6	4.2	2.2	3.3	−0.2	20.5
6	3.8	24.0	8.5	2.2	1.0	0.4	25.8

The superior and inferior GTV volumes may change up to 0.2 cm^3^ after deformable image registration in the ATS workflow comparing to the original GTVs. Average MUs were similar regardless of the workflow. The optimization time in the ATP workflow varied from <60 s (online) to ∼400 s (offline) and that for the ATS workflow varied from ∼300 s (online) to ∼900 s (offline).

#### 3.1.3 Adaptive patient plans for simulated daily setup variations

**FIGURE 5:**
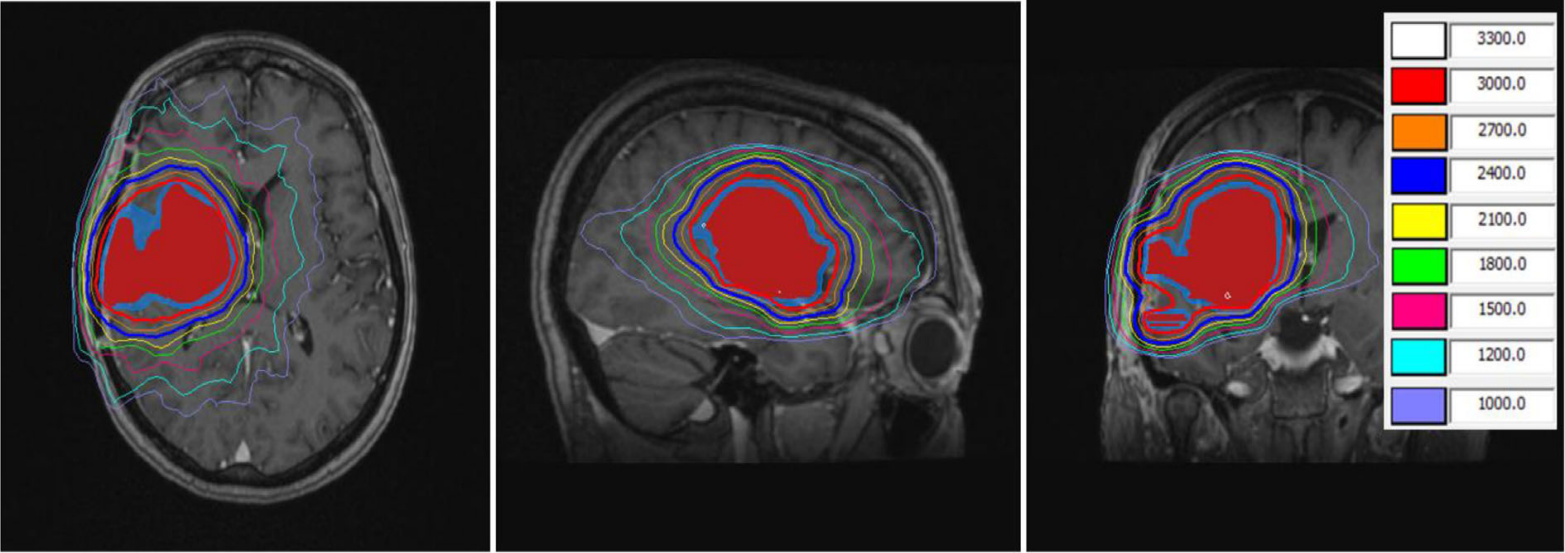
A patient magnetic resonance imaging (MRI) and Monaco reference plan. Red colorwash: GTV; blue colorwash: PTV. Isodose lines are in cGy

A patient MRI and Monaco reference plan is shown in Figure 5. The total PTV volume in the patient plan was 69.7 cm^3^. The primary lesion was 68.0 cm^3^, while a small lesion of 1.7 cm^3^ was located inferior of the primary lesion (Figure 5c, coronal view). The deformed volumes were verified for the ATS workflow in both tests within 0.1 cm^3^ of the volume in reference plan. The reference plan and adaptive plans were all normalized to achieve the same PTV coverage as in clinical plan. Comparison of the adaptive plans to the reference plan and clinical plan is shown in Table 3. The ATS workflow achieved better GI and PCI as well as brain V20. Adjacent brainstem's *D*_max_ was slightly higher in the ATS plans than in ATP plans, but still within tolerance (V30Gy ≤0.01 cm^3^), and was lower than that in the clinical plan. The GI, PCI, and brain V20 in the reference and adaptive plans were all inferior to those in clinical VMAT plan.

**TABLE 3:** Adaptive patient plan: Comparison of adapt‐to‐position (ATP) versus adapt‐to‐shape (ATS) workflows to reference and clinical plan

		**GI**	**PCI**	**Brainstem *D*_max_ (Gy)**	**Brain V20 (cm^3^)**
Reference plan		3.63	0.84	24.3	162.8
Clinical plan^a^		2.95	0.91	30.5	133.6
Adaptive test 1	ATP	3.52	0.83	25.1	160.9
	ATS	3.45	0.89	26.0	148.3
Adaptive test 2	ATP	3.6	0.83	25.0	163.6
	ATS	3.48	0.89	25.6	147.8

^a^ Clinical plan was generated in RayStation with VMAT technique.

### 3.2 End‐to‐end test

Repeated verification of MR image registration showed minor discrepancy (<0.5^o^ and <0.5 mm) between daily MR and verification MR after switching from the delineation insert to the chamber insert. The difference between the measured and planned doses was within 1% for both targets in the two treatment workflows, as shown in Table 4.

**TABLE 4:** Chamber measurements for daily adapt‐to‐shape (ATS) and adapt‐to‐position (ATP) plans on head phantom

	**ATP workflow**		**ATS workflow**	
	**Sup GTV**	**Inf GTV**	**Sup GTV**	**Inf GTV**
Adaptive plan (cGy)	2201.0	2263.0	2201.6	2205.6
Measurement (cGy)	2186.5	2255.0	2195.5	2194.0
% Difference	**0.7%**	**0.4%**	**0.3%**	**0.5%**

## 4 DISCUSSION

In this study, we demonstrated the feasibility of brain SRS on Elekta Unity MRL in regard to daily adaptive planning and delivery accuracy. The RTsafe head phantom with predefined brain metastatic lesions was used for planning and an end‐to‐end test. The main benefit of treating brain lesions on MR Linac is that we can clearly see the lesion on MR images and examine targets and OAR volumes for precise alignment and volume change evaluation, which potentially allows PTV margin reduction. Tumor volume may change between the time of simulation and treatment, as well as during the treatment course. The factors causing tumor volume change include (i) progressive tumor growth with time; (ii) response to radiation; (iii) response to concurrent chemotherapy; and (iv) volume change after surgery. With daily MR imaging on MRL that provides superior soft tissue visualization, these changes can be captured, and the treatment plan can be adjusted accordingly to avoid target miss or overdosing of OARs. Several clinical indications that could improve the outcomes for patients exist, and this includes for instance treatment of brain cavities or metastases with SRS. Cavities shrinkage or shape changes can occur over days and these can impact local control.^21^

Our observation showed up to 0.2 cm^3^ volume change for each of the GTVs in phantom study in the ATS workflow and only 0.1 cm^3^ difference on deformed GTV volumes in patient study. While the target volumes in the phantom and patient studies were unchanged in this study, these volume evaluation results conclude the overall errors of the imaging and deformable registration in the adaptation workflow. The detected small volume difference should be well covered by the PTV margin, which includes imaging, planning, and setup uncertainties. However, tumor volume change may be more complicated in real patient cases; thus, using contrast injection for pretreatment MR imaging is strongly recommended for brain metastasis, especially for smaller lesions and progressive disease.

We compared the two adaptation workflows that are available in the Elekta Unity MRL. From our observations, both ATP and ATS workflows can reasonably achieve clinical goals in daily adaptive planning. Compared with the ATP workflow, the ATS workflow is more robust to daily setup variations and is more consistent with the reference plan. The reason is that ATS workflow performs complete optimization of the machine parameters based on daily anatomy and allows adjustment of plan objectives, whereas the ATP workflow only adjusts the shape and weights of the original segments of the reference plan. The plan quality index of the ATS plans was within 5% in both phantom and patient plans. GI and V12 of the ATS plan were much lower than those of the ATP plan, and the PCI was higher than ATP plan for both preset translational shifts and random daily setup variation in phantom study. ATS workflow also allows better control of the OAR dosimetric constraints during plan reoptimization.

Normal brain dose is an important metric to evaluate the quality of adaptive plans in brain SRS. According to Limon et al., normal brain volume including target volumes, such as V12 for a single fraction and V20 for three fractions are associated with risk of symptomatic radionecrosis.^22^ Our phantom study showed that the ATP plans may result in significantly higher normal brain dose (up to 27%) relative to the reference plan. Conservative constraints on normal brain should be used in the reference plan to minimize the brain dose from the beginning.

In addition, the ATS workflow may be beneficial when rotational setup variation exists. Rotational setup variation may cause target coverage loss if it is not corrected before MRL treatment. Our phantom test showed a 5% reduction on GTV *D*_min_ with the ATP workflow if daily setup includes 3° pitch rotation. Extra attention is needed if rotational error exists when multiple lesions are treated with a single isocenter, and the ATS workflow is strongly recommended for this scenario in brain SRS treatment.

In our patient study, with the same target coverage, the brainstem maximum dose was lower in Monaco plan than that in clinical VMAT plan. However, the GI, PCI, and brain V20 were inferior for Monaco plan. Several studies reported similar results on the dosimetric comparison between Monaco IMRT plans and conventional IMRT/VMAT plans.^10^, ^23^, ^24^ While target coverage and critical OAR dose can be achieved comparably, the low dose volume was found to spread more for Monaco plans. Despite this, it was agreed that the clinical goals were still met in Monaco plans.^23^, ^24^ We will evaluate more patients in our dosimetric comparison study in the near future.

Online optimization with the ATS workflow takes longer time than the ATP workflow. The extra time in ATS workflow comes from (i) deformable image registration to propagate contours to daily MR image, (ii) contour review/adjustment by radiation oncologist, and (iii) plan optimization. In our phantom cases, the online plan optimization in ATS workflow took up to 5–10 min compared with <2 min for the ATP workflow. However, with the high prescription dose and IMRT technique used, the treatment time is normally much longer than this. The whole treatment time from imaging to completion of radiation delivery for 20 Gy of target dose in single fraction was observed to be around 40 min for ATP workflow and 50 min for ATS workflow. Several studied in the literature reported the ATS workflow to be about 15 min longer than ATP workflow.^25^, ^26^ Our phantom study showed less difference (∼10 min), because the lesions were smaller and we did not include any critical organs so the plan optimization was less challenging.

## 5 CONCLUSIONS

We demonstrated the feasibility of treating brain metastases SRS using the MR‐Linac with satisfactory dosimetric goals and delivery accuracy. We also showed that with online ATS plan adaptation, daily setup uncertainties could be better accounted for than with the simple ATP plan adaptation. Further the ATS plan can better maintain dosimetric goals for normal brain tissue, at the cost of longer plan adaptation time.

## CONFLICT OF INTEREST

The authors declare that there is no conflict of interest.

## AUTHOR CONTRIBUTIONS

Eun Young Han and He Wang conceived the project and drafted the manuscript. Eun Young Han, He Wang, Jinzhong Yang, and Pamela Castillo collected data, performed measurements, and analyzed the data. Tina Marie Briere, Debra Nana Yeboa, and Jinzhong Yang provided clinical expertise and supervision of the paper. Themistoklis Boursianis, Georgios Kalaitzakis, and Evangelos Pappas provided the head phantom and developed measurement insert. All authors reviewed, revised, and approved the manuscript.

## Data Availability

The data that support the findings of this study are available from the corresponding author upon reasonable request.
